# Cord blood 25(OH)D_3_, cord blood total immunoglobulin E levels, and food allergies in infancy: A birth cohort study in Chongqing, China

**DOI:** 10.1016/j.waojou.2022.100645

**Published:** 2022-04-01

**Authors:** Nian-Rong Wang, Shi-Jian Liu, Gui-Yuan Xiao, Hua Zhang, Yu-Jie Huang, Li Wang, Chun-Yan He

**Affiliations:** a Department of Children Healthcare, Chongqing Health Center for Women and Children, 120 Longshan Road, Yubei District, Chongqing, 400021, PR China; b Department of Clinical Epidemiology and Biostatistics, Children Health Advocacy Institute, Shanghai Children's Medical Center, Shanghai Jiao Tong University School of Medicine, 1678 Dongfang Road, Pudong New District, Shanghai, 200127, PR China

**Keywords:** Cord blood, Food allergy, Immunoglobulin E, 25-Hydroxy vitamin D_3_

## Abstract

**Background:**

Food allergy (FA) in infants has become a common disease worldwide. There are many controversies surrounding the relationships among levels of cord blood 25-hydroxy vitamin D_3_ [25(OH)D_3_], total immunoglobulin E (IgE), and FA.

**Methods:**

In this study, we recruited pregnant women in the third trimester undergoing obstetric examination in Chongqing City, Western China. Healthy full-term singleton births between May to August 2018 and November 2018 to January 2019 were included in the summer-birth and winter-birth cohorts, respectively. Questionnaires on vitamin D status in pregnancy and family allergies were used to investigate the pregnant women. The levels of <12 ng/mL, 12～20 ng/mL, and >20 ng/mL 25(OH)D_3_ in cord blood detected by liquid chromatography tandem mass spectrometry were considered deficient, insufficient, and sufficient, respectively. The electrochemiluminescence method was used to detect the total lgE levels in cord blood, classified into low-IgE (<0.35 IU/mL) and high-IgE (≥0.35 IU/mL) levels, respectively. Within postnatal 6 months, allergic symptoms in infants were investigated using questionnaire during the infants’ monthly physical examinations. Suspected cases of FA underwent a history inquiry, skin prick test, food elimination test, and open-food challenge for diagnosis of FA. Multivariate logistic regression was used to analyze the risk factors of FA in infants.

**Results:**

In this study, we recruited 741 pairs of pregnant women and infants, including 343 infants in the summer-birth cohort and 398 infants in the winter-birth cohort. The incidence of FA within postnatal 6 months was 6.88%, showing significantly higher incidence of FA in the winter-birth cohorts than in the summer-birth cohorts (10.3% vs. 2.9%, *χ*2 = 15.682, *P* = 0.000). Among the 741 infants, 47.1%, 27.5%, and 13.8% of infants had deficient, insufficient, and sufficient 25(OH)D_3_, respectively, in the cord blood; 81.5% and 18.5% of infants had total low-IgE and total high-IgE levels, respectively, in the cord blood. No significant correlation was found between the 25(OH)D_3_ and IgE levels (r = −0.038, *P* = 0.300). Logistic regression analysis showed that winter birth [odds ratio (OR) 95% confidence interval (CI): 4.292 (2.003～8.359)] compared with infants in summer birth group, and sufficient (>20 ng/mL) 25(OH)D_3_ levels in cord blood [OR (95% CI): 2.355 (1.129～4.911) compared with infants in the deficient group (<12 ng/mL) and 3.782 (1.680～8.514) compared with infants in the insufficient group (12～20 ng/mL)] were independent risk factors for FA in infants within postnatal 6 months.

**Conclusions:**

Winter birth and sufficient 25(OH)D_3_ levels in infant cord blood were independent risk factors for FA in infants. 25(OH)D_3_ and total IgE levels in cord blood cannot be used as predictors of FA in early infancy.

## Introduction

Food allergy (FA) among children is on the rise globally and has become a common public health problem. The occurrence and progression of FA may be related to changes in the environment and lifestyles in human populations.^1^ Identification of relevant predictive indicators for the early intervention of FA may help to reduce or alleviate the occurrence of FA in children.^2^

In recent years, the number of ecological and epidemiological studies on the relationship between the birth season and risk of FA has increased, with more studies showing the potential role of vitamin D in the occurrence of FA.^3^ Serum 25-hydroxy vitamin D_3_ [25(OH)D_3_] is the best indicator of vitamin D status in the body, reflecting the total intake of vitamin D formulations and foods containing vitamin D, and the level of vitamin D synthesized by the skin after sufficient sun exposure.^4^ Studies have shown that 25(OH)D_3_ may affect the occurrence of FA via the following mechanisms. First, it affects the function of macrophages, dendritic cells, B cells, T cells, and epithelial cells, regulating the function of target genes or epigenetic modifications and playing an important role in the innate and adaptive immune responses.^5^ Second, it passes to the fetus through the placenta and begins to exert immunomodulatory effects during the fetal period^6^ and may affect allergic diseases in the offspring, such as asthma and wheezing.^6^, ^7^, ^8^, ^9^ However, the existing research is inconclusive as to whether 25(OH)D_3_ is related to the occurrence of FA in offspring.^10^, ^11^, ^12^, ^13^, ^14^ It is worth investigating whether the examination of cord blood 25(OH)D_3_ levels helps predict the occurrence of FA.

Immunoglobulin (Ig)E is mainly produced by B lymphocytes. IgE does not pass through the placenta, in general. However, it can be detected in the cord blood of children with allergies, indicating that the fetus has been sensitized in the mother's body. Some scholars have proposed that high total IgE levels in cord blood may help predict the occurrence of allergic diseases in the offspring. However, it is still inconclusive as to whether the total IgE levels in cord blood help to predict the occurrence of FA in the offspring.^15^

The relationship between total IgE and vitamin levels in cord blood has been rarely reported. This study used a prospective birth cohort to follow up from the third trimester of pregnancy in the mother to 6-month-old infants. Through the detection of 25(OH)D_3_ and total IgE levels in cord blood, the longitudinal follow-up of FA in infants within postnatal 6 months was conducted, to explore the correlations among 25(OH)D_3_, total IgE levels in the cord blood, and FA in infants, providing clinical clues for the early prediction of FA.

## Methods

### Research design

In this study, we conducted a prospective birth cohort study to assess 25(OH)D_3_ and total IgE levels in the cord blood of full-term healthy newborns, and the occurrence of FA in infants within postnatal 6 months. Guardians of all selected participants provided written informed consent before participating in the study.

### Research participants

We recruited pregnant women in the third trimester undergoing obstetric examination. Healthy full-term singletons born between May to August 2018 and November 2018 to January 2019 were included in the summer-birth and winter-birth cohorts, respectively. Child health management was carried out in infants once a month until the age of 6 months. The exclusion criteria in this study were: (1) pregnant women with hypertension, diabetes, or other major physical and mental illness (eg, immunological disorders and infectious diseases) during pregnancy; and (2) newborns with unnatural conception (eg, from pregnancy through assisted reproductive technology and from multiple births), birth defects, or ischemic hypoxia.

The parents or guardians of all patients signed a written informed consent form before they were recruited. This study was approved by the ethics committee of the Chongqing Health Center for Women and Children (L2017018).

### Questionnaire-based assessment

A face-to-face survey was conducted during the late trimester of pregnancy and each pregnant woman was interviewed by an investigator at the obstetric clinic. The questionnaires included the following items: (1) the Vitamin D section — whether or not to take vitamin D supplements, the brand, the dosage of each time, the frequencies each day, when to start and stop taking, etc; (2) the dietary section — whether or not to eat foods rich in vitamin A, such as liver, fish, eggs, carrots, etc., how often to eat per week, how much to eat per meal, how often to eat per day, etc; and (3) the sun exposure section — the season, the form, quantity, and frequency of outdoor activities, whether or not to use of sunscreen or umbrella, etc.

Before the newborns were discharged from the hospital, mothers or their guardians were required to complete a questionnaire about the general condition of the newborn and an assessment form on the risk of allergies in the infant. The questionnaire about the general condition of the newborn included the gestational age at birth, birth weight, birth height, and head circumference of the infant; and the educational background, age, smoking history, and pet cohabitation of the infants’ parents or guardians.

The “Infant Allergy Risk Assessment Form” was provided by the Chinese National Maternal and Child Health Center, and it had been used and accepted in children's food allergy clinic in China.^16^^,^^17^ The Infant Allergy Risk Assessment Form was used to initially screen infants at high risk of allergy, which included 4 sections and 11 questions: genetic factors (family history of allergies refers to the occurrence of allergies in any first-degree relative, such as parents or siblings), childbirth and feeding factors, medications used during pregnancy, and environmental factors during pregnancy in the mother. Total scores ≤2, 3～5, and ≥6 in the assessment form were classified as indicating a low risk, moderate risk, and high risk of allergies, respectively, in the infants.

When we investigated a family history of allergies, every pregnant woman was asked to provide medical records of allergies of herself, her husband, and their previous diagnosis of their children diagnosed by gastroenterologists, allergy immunologists, otolaryngologists, ophthalmologists, dermatologists, and so on.

### Collection and testing of cord blood samples

Five milliliters of cord blood was collected and stored at −80°C for later use when the newborn's umbilical cord was broken. In this study, SCIEX Triple QuadTM 4500MD liquid chromatography tandem mass spectrometry (AB SCIEX, Framingham, MA, USA) was used to detect levels of 25(OH)D_3_, and the cobas e 601 electrochemiluminescence automated analyzer as well as an IgE (IgE II) detection kit (Roche, Basel, Switzerland) were used to detect total IgE levels in the cord blood samples.

### Determination of 25(OH)D_3_ and total IgE levels

In this study, the 25(OH)D_3_ levels in cord blood samples were classified into vitamin D deficiency (<12 ng/mL), vitamin D insufficiency (12～20 ng/mL), and vitamin D sufficiency (>20 ng/mL).^18^ According to studies in China and other countries, normal levels of total IgE in infant cord blood should be less than 0.5 IU/mL.^19^ This study defined the <0.35 IU/mL total IgE as low IgE level and ≥0.35 IU/mL total IgE in cord blood as high IgE in the cord blood.

### Food allergy diagnosis

**Fig. 1:**
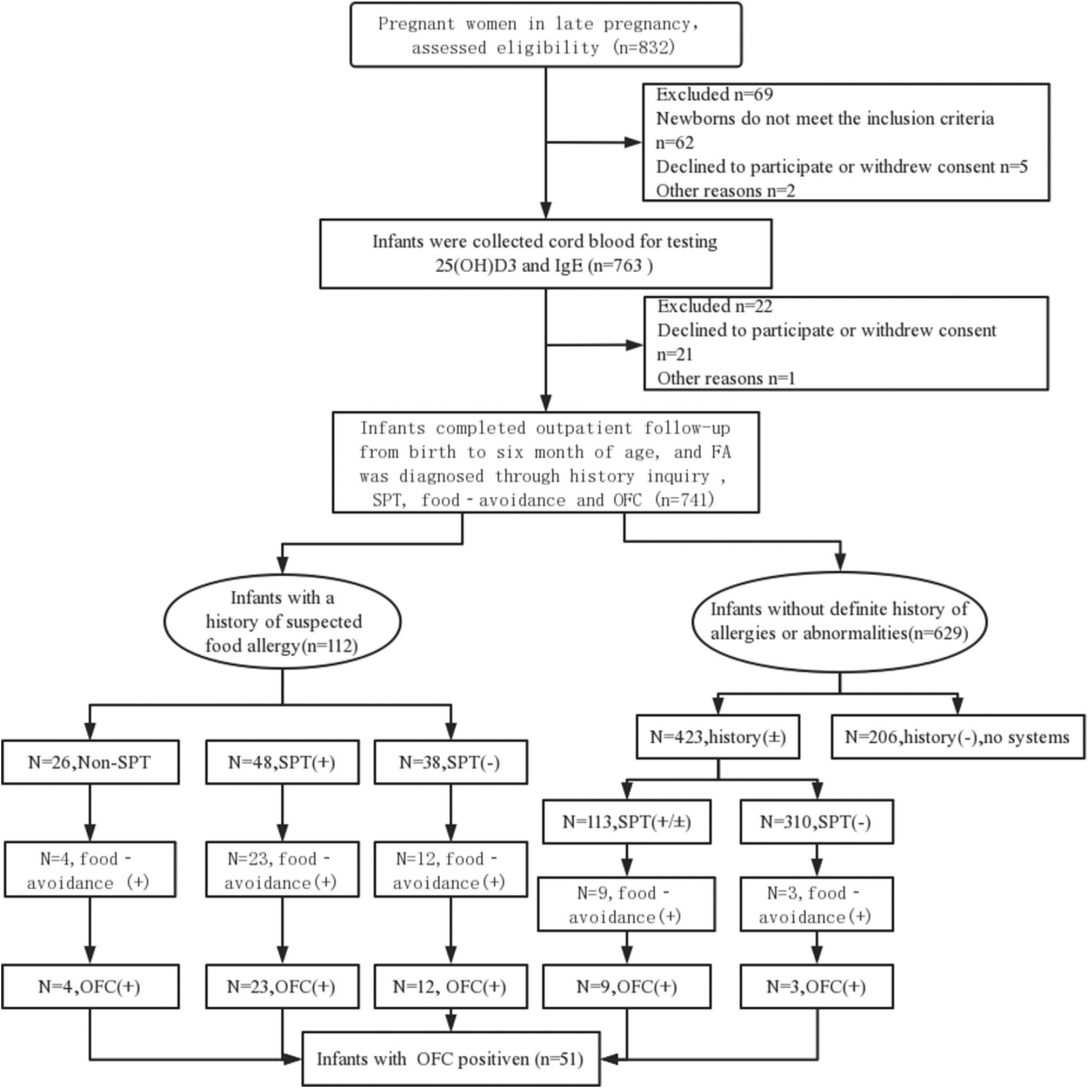
The consort study diagram

In the monthly outpatient follow-up at postnatal 6 months, the symptoms on infants' skin, in the digestive tract, respiratory tract, and other systems were carefully queried and recorded by physicians. When the medical history of an infant suggested the possibility of FA, a comprehensive physical examination, skin prick test (SPT), food elimination test, and open food challenge (OFC) were carried in sequence, according to the FA diagnostic procedure. FA was diagnosed when positive results of OFC were obtained. Once the diagnosis was confirmed, the above tests were terminated.^20^ In the SPT, allergen reagents and standard needles for skin prick (Merck K GaA, Darmstadt, Germany) were used in accordance with conventional operating procedures. Physiological saline was used as a negative control, and histamine was used as a positive control in the SPT. Wheal with a length >3 mm larger than the negative control reaction was considered a positive response to the food allergen which called food sensitization. In the food elimination test, suspected allergen foods were strictly avoided in the infant's diet for at least 2–4 weeks. During the same period, it was necessary to avoid infants taking medications that could potentially affect the immune system or mask the symptoms of FA. The result was considered positive if the previous symptoms improved or disappeared and negative if there was no change in the previous symptoms (Fig. 1).

For the OFC,^21^ a double-blind, placebo-controlled OFC is the gold standard for diagnosing FA. In this study, research participants were infants under 1 year of age. The results of OFC were hardly affected by psychological factors among our research participants, so the OFC could be used to obtain a definitive diagnosis. Inducement of previous symptoms was considered a positive OFC result, indicating a clear diagnosis of FA; otherwise, negative OFC results referred to a negative diagnosis of FA.

### Statistical analysis

Measurement data, such as levels of 25(OH)D_3_ and total IgE in the cord blood, are presented as mean (standard deviation). Count data, such as the incidence of FA, are presented as n (%). The chi-square test was used to compare the incidence of FA between different seasons, infant's allergy risk levels, family history of allergies, and different cord blood 25(OH)D_3_ groups and different cord blood total-IgE groups. The *t*-test was used to compare the mean (standard deviation) of cord blood 25(OH)D_3_ and total IgE between different seasons, infant's allergy-risk levels, and the presence or absence of family history of allergies or FA(s). Spearman's correlation was used to analyze the correlation between 25(OH)D_3_ and total IgE levels in cord blood. Late pregnancy period factors in the questionnaires, such as home address, household income, educational background, allergy history, delivery method, outdoor activities, UV protection, consumption of nutritional supplements, consumption of nutrient food, and other factors, were included in the univariate analysis of cord blood 25(OH)D_3_. Relevant indicators in the research questionnaires, such as birth season, sex, birth weight, and height of the infant; home address, household income, and educational background of the infant's parents; allergy-risk level, delivery method, UV protection during outdoor activities, consumption of nutritional supplements, and application of sunscreen during outdoor activities; pet cohabitation, cord blood 25(OH)D_3_ grouping, cord blood total IgE grouping, and other factors, were included in the univariate analysis of FA in infants. Subsequently, factors that showed statistical significance in the univariate analysis of FA were reentered in a stepwise regression analysis and multivariate logistic regression analysis of FA in infancy. All data were analyzed using IBM SPSS 25.0 statistical software (IBM Corp., Armonk, NY, USA). *P* < 0.05 was considered statistically significant.

## Results

### General condition of pregnant women and newborns

We initially recruited 832 pregnant women in late pregnancy, completed 6 months of infant follow-up with 741 mother-child pairs after delivery. Among the 741 pairs of mothers and infants, there were 343 summer-birth infants and 398 winter-birth infants, including 51.9% male and 48.1% female infants. The average gestational age of infants was 38.7 ± 1.3 weeks. Infants with a family history of allergies accounted for 23.5%, with the proportions of positive allergy history in the mother, father, and older siblings accounting for 12.6%, 10.8%, and 4.4%, respectively. The proportions of infants with a low risk, moderate risk, and high risk of allergies were 58.2%, 26.2%, and 15.5%, respectively. No significant differences in sex, birthweight, birth length, presence of family history of allergies, father's age, and educational background of the parents were found between the summer-birth and winter-birth infants. However, significant differences in average gestational age, mother's age, and infant's allergy-risk classification were found between summer-birth and winter-birth infants (Table 1).

**Table 1:** Baseline demographics of summer-birth and winter-birth infants.

Variables	All	Summer- birth	Winter-birth	t/*χ*^2^-value	*P*-value
Infant sex					
Male, n (%)	385 (51.9)	181 (52.70)	204 (51.3)	0.205	0.651
Female, n (%)	356 (48.1)	162 (47.30)	194 (48.7)		
Infant's birth weight (g) (Mean ± SD)	3309.9 ± 430.0	3299.5 ± 423.1	3318.9 ± 436.2	−0.612	0.540
Infant's birth length (cm) (Mean ± SD)	49.8 ± 1.8	49.9 ± 1.8	49.8 ± 1.9	0.415	0.680
Gestational weeks (Mean ± SD)	38.7 ± 1.3	38.8 ± 1.3	38.6 ± 1.2	2.308	0.021
Family history of allergies in infants					
Yes, n (%)	170 (23.5)	86 (26.5)	84 (21.1)	2.934	0.087
No, n (%)	552 (76.5)	238 (73.5)	317 (78.9)		
Delivery method					
Vaginal delivery, n (%)	384 (51.9)	200 (58.3)	184 (46.2)	11.054	0.001
Cesarean section, n (%)	357 (48.1)	143 (41.7)	214 (53.8)		
Father's age (years) (Mean ± SD)	32.1 ± 5.0	31.8 ± 4.5	32.4 ± 5.4	−1.851	0.065
Mother's age (years) (Mean ± SD)	30.2 ± 4.2	29.8 ± 3.9	30.5 ± 4.4	−2.329	0.020
Mother's education					
Bachelor's degree or below, n (%)	323 (43.6)	138 (40.2)	185 (46.5)	2.926	0.087
Above bachelor's degree, n (%)	418 (56.4)	205 (59.8)	213 (53.5)		
Father's education					
Bachelor's degree or below, n (%)	321 (43.3)	135 (39.4)	186 (46.7)	3.786	0.052
Above bachelor's degree, n(%)	420 (56.7)	208 (60.6)	212 (53.3)		
Allergy history in mother					
Yes, n (%)	91 (12.6)	47 (14.5)	44 (11.1)	4.368	0.113
No, n (%)	631 (87.4)	277 (85.5)	354 (88.9)		
Allergy history in father					
Yes, n (%)	78 (10.8)	42 (17.8)	36 (9.0)	3.334	0.189
No, n (%)	644 (89.2)	282 (82.2)	362 (91.0)		
Allergy history in infant's older sibling(s)					
Yes, n (%)	32 (4.4)	12 (3.7)	20 (5.0)	1.538	0.463
No, n (%)	690 (95.6)	312 (96.3)	378 (95.0)		
Infant's allergy-risk classification					
Low risk, n (%)	424 (58.2)	199 (60.3)	225 (56.5)	7.091	0.029
Moderate risk, n (%)	191 (26.2)	72 (21.8)	119 (29.9)		
High risk, n (%)	113 (15.5)	59 (17.9)	54 (13.6)		

SD, Standard deviation

### Occurrence of food allergy in infants

Among the 741 infants, 112 infants had suspected food allergy history, 86 of whom completed SPT. Among 86 infants, 23 of 48 infants with SPT positive were OFC positive, and 12 of 39 infants with SPT negative were OFC positive. Furthermore, 4 of 26 infants without SPT result were OFC positive. Four hundred twenty-three infants with symptoms that did not seem to have anything to do with food underwent SPT, 113 of whom were SPT positive and 12 infants with positive OFC results. For the remaining 206 infants without suspicious symptoms there was no need to do SPT, food-avoidance diets, or OFC. As a result, a total of 51 infants were diagnosed with food allergy, 32 (62.75%) were IgE mediated, and 19 (37.25%) were non-IgE mediated (Fig. 1).

The incidence of FA within postnatal 6 months among the 741 infants was 6.88% (51/741), with incidences of FA in male and female infants of 7.3% (28/385) and 6.5% (23/355), respectively (*χ*2 = 0.181, *P* = 0.67). Among them, 51 infants were diagnosed with food allergies: cow's milk allergy 2.43% (18/741), egg yolk allergy 2.29% (16/741), egg white allergy 0.13% (1/741), wheat allergy 0.27% (2/741), soy allergy 0.27% (2/741), shrimp allergy 0.13% (1/741), peanut allergy 0.13% (1/741), cow's milk, egg yolk and shrimp allergy 0.54% (4/741), egg yolk and shrimp allergy 0.13% (1/741), egg yolk and peanut allergy 0.4% (3/741), cow's milk and egg yolk allergy 0.13% (1/741), and cow's milk and wheat allergy 0.13% (1/741).

Among the infants with FA within postnatal 6 months, 62.75% (32/51) of infants had allergy symptoms of the skin, such as eczema and wheal; 37.25% (19/51) of infants had gastrointestinal symptoms, such as hematochezia and spitting up.

The incidence of FA was lower in summer-birth infants than in winter-birth infants (2.9% vs. 10.3%, *χ*2 = 15.682, *P* = 0.000). The incidence of FA among infants in the low-risk, moderate-risk, and high-risk groups was 6.2%, 8.4%, and 7.1%, respectively (*χ*2 = 1.010, *P* = 0.603). The incidence of FA in the negative-allergy and positive-allergy family history groups was 7.6% and 5.3%, respectively (*χ*2 = 0.869, *P* = 0.227; Table 2).

**Table 2:** Incidence of food allergy in infants within postnatal 6 months (%)

Classification	n (%)	*χ*^2^	*P*-value
Seasons			
Summer-birth	10 (2.9)	15.682	<0.001
Winter-birth	41 (10.3)		
Infant's allergy-risk classification			
Low risk	27 (6.2)	1.010	0.603
Moderate risk	16 (8.4)		
High risk	8 (7.1)		
Family history of allergies			
No	42 (7.6)	0.869	0.227
Yes	9 (5.3)		

### Levels of 25(OH)D_3_ and total IgE in cord blood

**Fig. 2:**
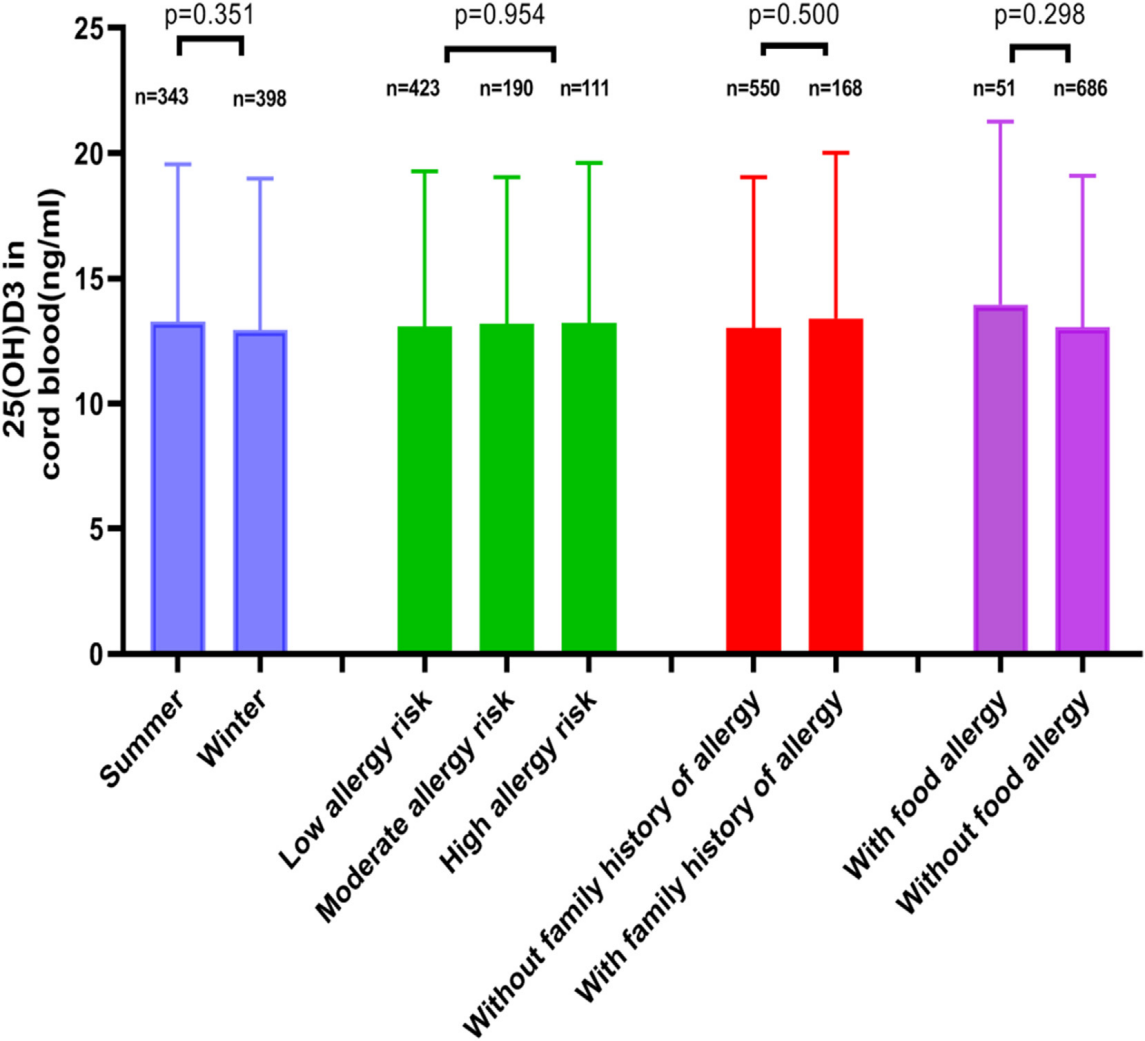
Comparison of mean values of 25(OH)D_3_ in cord blood among different groups

**Fig. 3:**
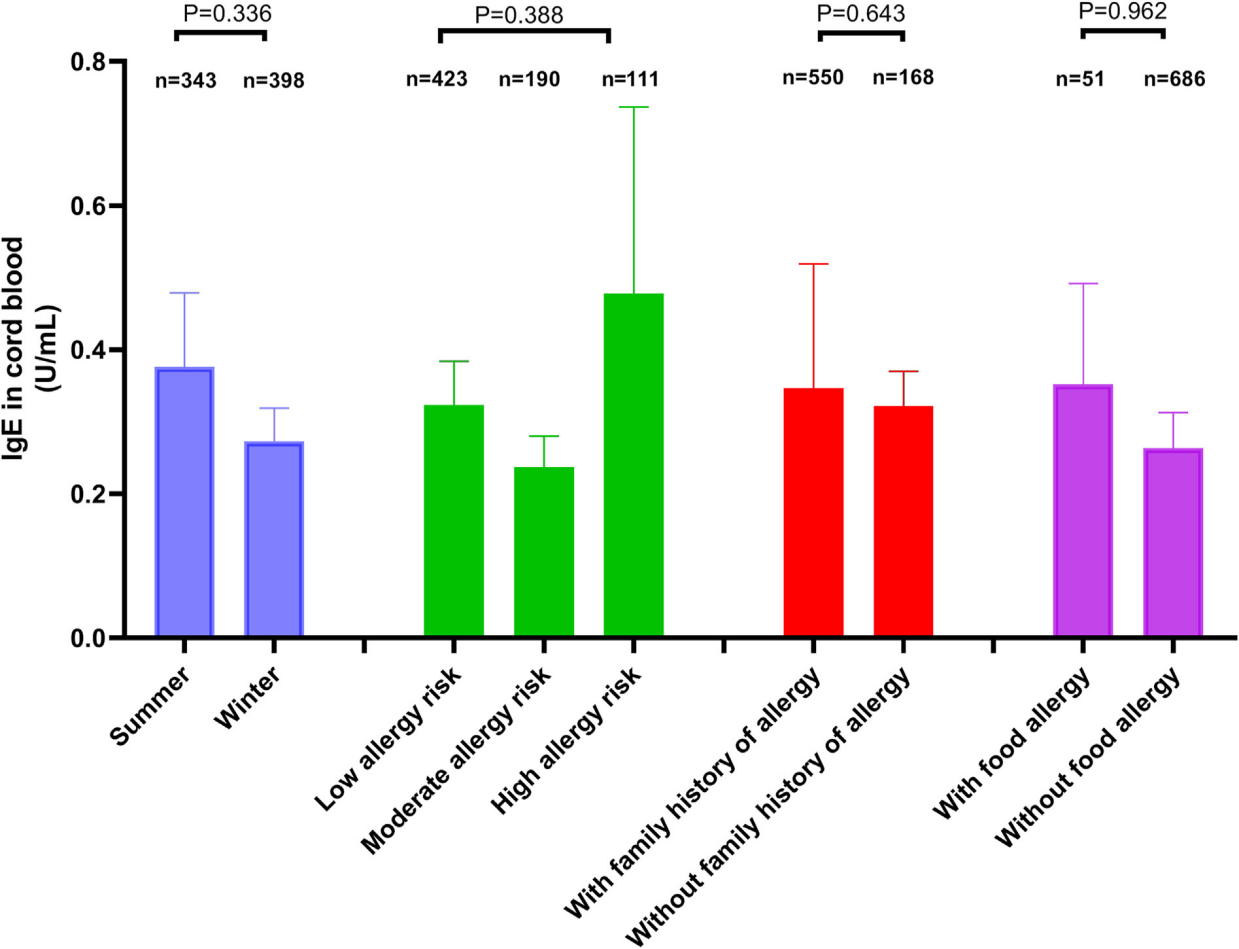
Comparison of mean values of total IgE in cord blood among different groups

The mean value of cord blood 25(OH)D_3_ in infants was 13.10 ± 6.15 ng/mL (ranging from 2.7 to 40.8 ng/mL). According to the grouping of infants by birth season, allergy-risk, and family history of allergies, no statistical differences in the mean value of cord blood 25(OH)D_3_ were found between summer-birth and winter-birth infants, among the 3 allergy-risk groups, or between infants with and without a family history of allergies (all *P* > 0.05; Fig. 2). Among the 741 infants, the mean value of cord blood total IgE was 0.32 ± 1.45 U/mL (ranging from 0.01 to 28.7 U/mL). No significant differences in cord blood total IgE were found between summer-birth and winter-birth infants, among the 3 allergy-risk groups, or between infants with and without a family history of allergies (all *P* > 0.05; Fig. 3). The mean value of total IgE in the cord blood of male infants was significantly higher than that of female infants (*t* = 2.494, *P* = 0.013). Spearman's rank correlation analysis showed no significant correlation between cord blood total IgE and the cord blood 25(OH)D_3_ (*r* = −0.038, *P* = 0.300).

### Cord blood 25(OH)D_3_ and total IgE and food allergy in infants

No significant differences in mean values of cord blood 25(OH)D_3_ and mean values of cord blood total IgE were found between infants with FA and infants without FA (*t* = 1.041, *P* = 0.298; *t* = −0.047, *P* = 0.962; Fig. 3). According to the measured values of cord blood 25(OH)D_3_, the infants were divided into a 25(OH)D_3_-deficient group (47.1% [343/741]), 25(OH)D_3_-insufficient group (27.5% [300/741]), and 25(OH)D_3_-sufficient group (13.8% [94/741]), with incidences of FA 7.0%, 4.7%, and 13.8%, respectively and a significant difference in the incidence of FA among the 3 groups (*χ*2 = 9.336, *P* = 0.009). No significant difference in the incidence of FA was found between the 25(OH)D_3_-deficient and -insufficient groups (*χ*2 = 1.563, *P* = 0.139), but significant differences in FA incidence existed between the 25(OH)D_3_-insufficient and -sufficient groups (*χ*2 = 9.415, *P* = 0.004) and between the 25(OH)D_3_-deficient and -sufficient groups (*χ*2 = 4.445, *P* = 0.033). According to measured values of cord blood total IgE, the infants were divided into a low-IgE group and high-IgE group, with incidences of FA 7.0% and 6.6%, respectively, but with no significant difference in the incidences of FA between the two groups.

### Multivariate logistic regression analysis of food allergy

In a univariate analysis of factors affecting cord blood vitamin D, egg yolk, calcium, and multinutrient combinations were associated with the level of cord blood vitamin D, but probiotic intake and other factors were not (*P* = 0.212) (Table 3).

**Table 3:** Univariate analysis of the influencing factors of cord blood vitamin D.

Variables	Number of cases	Mean ± SD	Standard error	F	*P*
Supplementation of egg yolk					
Daily	357	13.771 ± 5.892	0.311	4.864	0.002∗
Often	219	12.688 ± 6.356	0.429		
Occasionally	96	11.308 ± 6.176	0.630		
Rarely or not	49	13.971 ± 6.276	0.896		
Supplementation of multivitamins					
Daily	565	14.099 ± 6.029	0.253	25.279	<0.001∗
Often	38	10.536 ± 5.260	0.853		
Occasionally	38	8.147 ± 4.505	0.730		
Rarely or not	83	9.895 ± 5.394	0.592		
Supplementation of calcium					
Daily	625	13.639 ± 6.024	0.240	11.176	<0.001∗
Often	50	9.412 ± 5.986	0.846		
Occasionally	24	9.791 ± 4.853	0.990		
Rarely or not	25	10.672 ± 6.600	1.320		
Supplementation of probiotics					
Daily	23	15.083 ± 5.591	1.165	1.504	0.212
Often	32	14.578 ± 5.527	0.977		
Occasionally	41	12.929 ± 5.978	0.933		
Rarely or not	628	12.984 ± 6.140	0.228		

∗*P* < 0.05; SD, Standard deviation; Often, once in about 3–5 weeks; Occasionally, once in about 3–5 weeks; Rarely or not, once in about 3–5 months or hardly ever

The other univariate analysis showed that the birth season, application of sunscreen during outdoor activities, frequency of calcium supplementation, main source of household income, and cord blood 25(OH)D_3_ grouping were risk factors for the incidence of FA in infancy (*P* < 0.1).

Multivariate logistic regression analysis showed that compared with summer-birth infants, the odds ratio (OR) and 95% confidence interval (CI) of the occurrence of FA in winter-birth infants was 4.292 (2.003～8.359). In comparison with infants in the 25(OH)D_3_-deficient group (<12 ng/mL) or infants in the 25(OH)D_3_-insufficient group (12～20 ng/mL), the OR (95% CI) of the occurrence of FA in 25(OH)D_3_-sufficient group (>20 ng/mL) was 2.355 (1.129～4.911) and 3.782 (1.680～8.514), respectively (Table 4).

**Table 4:** Multivariate logistic regression analysis of food allergy in infancy.

Variables	Regression coefficient	Standard error	Wald *χ*^2^	*P*-value	OR (95% CI)
Birth season					
Winter birth vs. summer birth	1.409	0.364	14.950	**< 0.001**	4.09 (2.00–8.36)
Cord blood 25(OH)D_3_					
Sufficiency vs. deficiency	0.857	0.375	5.218	**0.022**	2.36(1.13–4.91)
Sufficiency vs. insufficiency	1.330	0.414	10.323	**0.001**	3.78(1.68–8.51)

Note: OR refers to the unadjusted odds ratio value. CI, confidence interval; 25(OH)D3, 25-hydroxy vitamin D3

## Discussion

Vitamin D has an extensive range of immunomodulatory effects, but the relationship between vitamin D and FA is still inconclusive.^22^ Total IgE level in cord blood as a predictor of allergic diseases has aroused extensive attention.^23^ IgE antibodies are markers for sensitization in the fetus, and the detection rate of food allergen IgE antibodies in cord blood is often higher than that of inhaled allergen IgE antibodies, suggesting that cord food IgE level may be used as a predictor for FA; however, the research results in recent years are inconsistent.^23^^,^^24^

In this study, the incidence of FA in infants within postnatal 6 months was 6.88%, with incidences of 10.3% in winter-birth infants and 2.92% in summer-birth infants, showing significant differences by birth season. In addition, the incidence of FA in winter-birth infants was 4.29 times that of summer-birth infants. These results suggested that the occurrence of FA in infants is significantly affected by the seasonal environment. Thus, it is necessary to focus on allergy prevention for children born in the winter. The Health Nuts study in Australia has shown that children born in the summer are 55% less likely to have FA than those born in other seasons.^25^ Mullin et al confirmed that the proportion of FA in children born during the winter and fall, and during the summer and spring, accounted for 57% and 43%, respectively,^26^ which is consistent with our findings. This may be related to the impact of birth season on the neonate's immune cells and functions, thereby affecting the subsequent risks of immune-related diseases.^27^ Summer-birth infants have the lowest levels of all immune cells and cell mediators, showing immune quiescence; winter-birth infants have the highest levels of innate immune cells, activated T cells, as well as IL-5, IL-1β, IL-17A, and IL8. This condition of the immune system can easily lead to subsequent allergy.^26^

In this study, we found no differences in the mean value of cord blood 25(OH)D_3_ between summer-birth and winter-birth infants, indicating that the season had no effect on cord blood 25(OH)D_3_ levels. One possible reason maybe that many pregnant women in the main urban area of Chongqing take daily 600 IU vitamin D supplements during pregnancy and pay greater attention to daily outdoor activities to minimize the differences in vitamin D synthesis caused by differences in ultraviolet rays between winter and summer.^18^ In addition, the cord blood 25(OH)D_3_ levels in this study did not differ among different allergy-risk levels or between the presence or absence of a family history of allergies, suggesting that cord blood 25(OH)D_3_ levels were not significantly affected by genes associated with allergies.

Our study also showed that 25(OH)D_3_ deficiency in the cord blood of full-term healthy newborns in Chongqing, China accounted for 47.1% of infants whereas 27.5% of infants had 25(OH)D_3_ insufficiency and 13.8% had 25(OH)D_3_ sufficiency; in other words, 86.2% of newborns had a low level of 25(OH)D_3_ in the cord blood, which is consistent with the findings of other studies.^28^, ^29^, ^30^, ^31^ This suggests that low vitamin D levels in cord blood are more common in healthy newborns. Low levels of 25(OH)D_3_ in cord blood may increase the risk of FA. Mullin et al. showed that a sufficient level of cord blood 25(OH)D_3_ (30～40 ng/mL) resulted in a low risk of peanut allergy by 6 years of age. A study from Taiwan showed that cord blood 25(OH)D_3_ levels are inversely related to sensitization to cow's milk in 2-year-old infants.^32^ Continuously insufficient 25(OH)D_3_ levels during the neonatal period and infancy likely causes FA in infants and young children.^33^ However, Yepes-Nunez et al reviewed studies on vitamin D supplementation in pregnant women, breastfeeding women, and infants and showed that, regardless of the different periods, supplementation with vitamin D had no clear preventive effects on FA.^34^^,^^35^ Other birth cohort studies have shown that there is no correlation between cord blood 25(OH)D_3_ deficiency and FA at the ages of 1, 2, or 5 years.^30^^,^^36^^,^^37^ There may be a U-shaped relationship between cord blood 25(OH)D_3_ deficiency and FA.^38^^,^^39^

This study showed that the risk of FA in infants with sufficient cord blood 25(OH) D_3_ (>20 ng/mL) was 2.36 times and 3.78 times that of infants with deficient and insufficient cord blood 25(OH)D_3_, respectively, with no significant difference between the deficient and insufficient groups. These results suggested that sufficient cord blood 25(OH) D_3_ level is an independent risk factor for FA in infancy. High levels of 25(OH)D_3_ at birth and in early infancy may increase the risk of FA at 3 years of age. The possible mechanism may be that the epigenetic imbalance of thymic stromal lymphopoietin is involved in the programming of vitamin D-related allergic diseases.^40^ A study from Germany also showed that the correlation between pregnancy and cord blood 25(OH)D_3_ levels reached as high as 0.8, and cord blood 25(OH)D_3_ levels were positively correlated with the occurrence of FA by 2 years of age, which may be related to a high level of 25(OH) D_3_ leading to the inability to exert immune tolerance, thereby causing FA.^41^ Thus, routine supplementation of vitamin D is not recommended to prevent FA. Previous studies have shown that total IgE antibodies in cord blood have a short-term predictive effect on asthma or sensitization, but the results of long-term predictive effects are contradictory.^42^ A study by Nissen et al showed that the coexistence of a family history of allergies and an increase in total IgE in the cord blood may be related to the occurrence of FA by age 1.5 years. However, the total IgE level in cord blood or a family history of allergies alone cannot be used to predict sensitization or the incidence of allergic diseases, such as FA.^23^ In this study, the total IgE levels in cord blood were low, with 81.5% of infants having a low level (<0.35 IU/mL). No significant differences in total IgE levels in the cord blood were found, regardless of whether infants had a family history of allergies, had different allergy-risk levels, had or did not have FA, and whether infants were born in the summer or winter. This suggests that intrauterine sensitization did not induce higher incidence of FA in infants with a positive allergy history or those with a high risk of allergies, which is consistent with Hatixhe's research.^43^ In this study, total IgE levels in the cord blood were correlated with the infant's sex, indicating a higher level of total IgE in the cord blood of male than in female infants, which is also consistent with the findings in another study in which sex was an independent risk factor for increased total IgE levels in cord blood.^44^ However, no significant difference in the incidence of FA was found between sexes, suggesting that high levels of total IgE in the cord blood of male infants cannot be used to predict the occurrence of FA in male infants.

Studies have shown that the relationship between 25(OH)D_3_ levels and total IgE levels in the whole population or in infants or childhood is complicated, showing different conclusions such as negative correlation, U-shaped correlation, and irrelevance.^45^ However, few studies have revealed the relationship between 25(OH)D_3_ and total IgE in cord blood. This study showed that cord blood 25(OH)D_3_ was not significantly related to total IgE in cord blood. No significant differences in 25(OH)D_3_ levels and total IgE levels in cord blood were found between infants with and without FA, suggesting that 25(OH)D_3_ and total IgE levels in cord blood cannot be used as predictors of FA in early infancy.

In summary, this study showed that winter birth was an independent risk factor for FA in infancy, and deficient or insufficient 25(OH)D_3_ levels was common and was a protective factor for the risk of FA in infancy. Total IgE levels in cord blood were generally low, which was unrelated to cord blood 25(OH)D_3_ levels or with the occurrence of FA in early infancy. Neither of these 2 indexes could effectively predict the occurrence of FA in early infancy, and there was no need for routine testing and high-dose vitamin D supplementation to prevent FA in infants.

### Advantages and limitations

Given that this was a prospective birth cohort study with follow-up from the third trimester of pregnancy to infant age 6 months, we adopted a standardized diagnostic process for FA to ensure reliability of the research results using the medical history, allergen screening (SPT), food elimination test, and OFC. However, this study also has limitations: (1) the follow-up period was only half of a year, which may not fully reflect the relationship between 25(OH)D_3_ and total IgE levels in the cord blood and the occurrence of FA in later stages of childhood. Thus, a continued follow-up study is required; (2) we did not measure immune components in the cord blood, such as regulatory T cells, or immune cytokines such as interleukin-4 (IL-4), interleukin-5(IL-5), interleukin-13(IL-13), and interleukin-17(IL-17), making it impossible to reveal possible immune mechanisms of 25(OH)D_3_ and total IgE in cord blood that affect FA.

## Abbreviations

FA, Food allergy; 25(OH)D3, 25-hydroxy vitamin D3; IgE, Immunoglobulin E; SPT, Skin prick test; OFC, Open food challenge; FET, Food elimination test; IL-4, Interleukin-4; IL-5, Interleukin-5; IL-13, Interleukin-13; IL-17, Interleukin-17.

## Funding

This study was supported by the Chongqing Municipal Health Commission (2017HBRC017, 2018MSXM067).

## Availability of data and materials

The datasets used or analyzed during the current study are available from the corresponding author on reasonable request.

## Author contributions

Nian-Rong Wang, Chun-Yan He and Yu-Jie Huang contributed to the study implementation. Gui-Yuan Xiao contributed to the statistical plan. Nian-Rong Wang and Shi-Jian Liu analyzed the data and prepared study results. All co-authors contributed to the interpretation of findings. All co-authors contributed to revising the manuscript and approved the final version.

## Declaration of competing interests

All authors declare that they have no competing interests or financial disclosures.

## Ethics approval and consent to participate

The parents or guardians of all patients signed a written informed consent form before they were recruited. This study was approved by the ethics committee of Chongqing Health Center for Women and Children (L2017018).

## Declaration of publication

All authors declare that they consent to publication.
